# Incorporating Measures of Structural Racism into Population Studies of Reproductive Health in the United States: A Narrative Review

**DOI:** 10.1089/heq.2020.0081

**Published:** 2021-02-25

**Authors:** Julianna G. Alson, Whitney R. Robinson, LaShawnDa Pittman, Kemi M. Doll

**Affiliations:** ^1^ Department of Obstetrics and Gynecology, University of Washington School of Medicine, Seattle, Washington, USA.; ^2^ Department of Epidemiology, UNC Gillings School of Public Health, University of North Carolina at Chapel Hill, Chapel Hill, North Carolina, USA.; ^3^ Lineberger Comprehensive Cancer Center, University of North Carolina at Chapel Hill, Chapel Hill, North Carolina, USA.; ^4^ Carolina Population Center, University of North Carolina at Chapel Hill, Chapel Hill, North Carolina, USA.; ^5^ Department of American Ethnic Studies, University of Washington, Seattle, Washington, USA.

**Keywords:** reproductive health, population health, African Americans, racism

## Abstract

**Purpose:** Black women in the United States face poor outcomes across reproductive health measures—from pregnancy outcomes to gynecologic cancers. Racial health inequities are attributable to systemic racism, but few population studies of reproductive health outcomes integrate upstream measures of systemic racism, and those who do are limited to maternal and infant health outcomes. Advances in understanding and intervening on the pathway from racism to reproductive health outcomes are limited by a paucity of methodological guidance toward this end. We aim to fill this gap by identifying quantitative measures of systemic racism that are salient across reproductive health outcomes.

**Methods:** We conducted a review of literature from 2000 to 2019 to identify studies that use quantitative measures of exposure to systemic racism in population reproductive health studies. We analyzed the catalog of literature to identify cohesive domains and measures that integrate data across domains. For each domain, we contextualize its use within population health research, describe metrics currently in use, and present opportunities for their application to reproductive health research.

**Results:** We identified four domains of systemic racism that may affect reproductive health outcomes: (1) civil rights laws and legal racial discrimination, (2) residential segregation and housing discrimination, (3) police violence, and (4) mass incarceration. Multiple quantitative measures are available for each domain. In addition, a multidimensional measure exists and additional domains of systemic racism are salient for future development into distinct measures.

**Conclusion:** There are quantitative measures of systemic racism available for incorporation into population studies of reproductive health that investigate hypotheses, including and beyond those related to maternal and infant health. There are also promising areas for future measure development, such as the child welfare system and intersectionality. Incorporating such measures is critical for appropriate assessment of and intervention in racial inequities in reproductive health outcomes.

## Introduction

Black women in the United States face poor outcomes across nearly all domains of reproductive health—from sexually transmitted disease to pregnancy to gynecologic cancers.^1^ Rigorous investigation of racial health inequities requires an analysis of racism,^2–4^ which is well recognized as a fundamental cause and social determinant of racial health inequities.^3,5–8^ Racism has adversely affected Black women's health since the enslavement of African people in North America.^9,10^ Black women have been advocating for autonomy, dignity, and rights in reproductive health for as long.^11^ Thanks to this long-standing labor, the clinical reproductive health field recognizes the role of racism as a structural determinant of health.^12^ While racism manifests at the institutional, interpersonal, and internalized levels,^2^ systemic racism refers to the totality of constantly reconstituting and reinforcing social and political systems and their policies, practices, and ideologies that engender inequities.^4,5^

Yet, few public health studies incorporate measures of systemic racism.^13^ Health research has historically been predominated by a conceptualization of race as an individual-level genetic trait or a population-level confounder rather than a rough proxy for risk of exposure to the mechanisms of social stratification in a race-conscious society.^14,15^ When racism has been incorporated, it is primarily in regard to interpersonal racial discrimination.^16^ In reproductive health, studies incorporating any level of racism have primarily focused on the outcomes of preterm birth, low birth weight, and infant mortality. Emerging research in maternal and infant health incorporates some measures of structural racism.^17–19^ While these are significant contributions, there is a need for population reproductive health studies—beyond perinatal outcomes—to include measures of systemic racism to identify underlying mechanisms of outcomes among Black women, and to provide the evidence base for effective intervention.

There is limited methodological guidance toward this goal. Gee and Ford^4^ describe approaches to conceptualizing structural racism. Groos et al.^20^ operationalize selected concepts for general health research, yet call for further elucidation of these measures specific to subdisciplines. To our knowledge, efforts in reproductive health have been limited to conceptual frameworks focusing on maternal health, infant health, and HIV/sexually transmitted diseases (STDs).^10,21^ The goal of this narrative review is to fill this gap by describing quantitative measures of exposure to systemic racism that can be applied in population-based studies of reproductive health.

### Definition of reproductive health

We consider reproductive health to comprise all clinical, quality of life, and health service outcomes related to fertility management and childbearing, menstruation and menopause, benign and malignant gynecologic conditions, and sexual functioning. Our approach to reproductive health is indebted to the Reproductive Justice movement, a grassroots movement for the rights and health of women of color, which was founded in the mid-1990s.^11^ Accordingly, we place reproductive health within the sociopolitical context of interlocking systems of power and oppression and recognize that freedom from these systems is critical for realizing reproductive justice.

## Methods

Three conceptual frameworks guide our review. The Ecosocial Theory guides us toward literature that considers disease process as the embodiment of socioecological context—including social inequity, resource deprivation, and societal-level and historical trauma.^22^ Fundamental Cause Theory guides selection of measures that give rise to differential access to “flexible resources” that operate along “replaceable pathways” from racism to health.^5,23^ Public Health Critical Race Praxis is the methodological approach to our work.^24^ Its tenets provide the framework by which we critically review the literature.

Using these frameworks, we conducted a review of literature from 2000 to 2019 to identify studies that use quantitative measures of exposure to systemic racism in population reproductive health studies. We first examined reproductive health literature and then broadened to general population health research. We excluded any study that did not include measures at the structural or systemic level. We cataloged each measure, its application, and available data sources. We then analyzed the catalog of literature to identify cohesive domains and measures that integrate data across domains. For each domain, we contextualize its use within population health research. We then describe metrics currently in use and opportunities for their application to reproductive health research. Given limited evidence on the topic, we make conceptually founded bridges between bodies of evidence. Finally, we discuss potential measures for future development.

## Results

### Domains

We identified four prominent domains of systemic racism that may affect reproductive health outcomes: (1) civil rights laws and legal racial discrimination, (2) residential segregation and housing discrimination, (3) police violence, and (4) mass incarceration. Table 1 aligns markers of exposure to structural racism with measures for inclusion in research.

**Table 1.** Recommended Measures for Inclusion in Population-Based Studies of Reproductive Health in the United States

Domain	Construct measured	Measure	Novel reproductive health applications
Civil Rights laws and legal racial discrimination	Legal and regulatory determinants of racial discrimination	Construction of cohorts in survey or administrative databases based on: - 1964 passage of the U.S. Civil Rights Act. - Designation of Jim Crow status of area of residence. - Age-period-birth cohorts based on Jim Crow status of area of residence and passage of U.S. Civil Rights Act. - Timing and geography of other federal and state legislation or enforcement rules/regulations	Quality of contraceptive care Hysterectomy rates (use and overuse) Transgenerational epigenetic stress mechanisms underlying gynecologic disease
Residential Segregation and Housing Discrimination	Spatial polarization by race and income	Index of Concentration at the Extremes	Quality of management of gynecologic pain Care delay for benign or malignant gynecologic disease
	Composite resource deprivation	Area Deprivation Index	
	Denial of home ownership in particular areas	Redlining Index	Quality of treatment for gynecologic cancer Use of hysterectomy alternatives for uterine fibroids
	Denial of financial resources for home ownership	Racial Bias in Mortgage Lending Index	
Police Violence	Individual or community-level exposure to the fatal or nonfatal violence of policing	- Estimation of rates of police killings by geography, based on validated data sources. - Use of data from Survey of Police-Public Encounters. - Inclusion of Jackson, Hogue, Philips contextualized stress measure police violence items in survey research	Physiologic stress mechanisms underlying gynecologic disease severity (fibroids, endometriosis, and infertility). Care delay for gynecologic cancer due to restricted mobility and/or fear of criminalization
Mass Incarceration	Individual or community-level exposure to incarceration	No established measures. - Individual exposure to incarceration using Public Use Microdata Samples. - Estimation of population rates of incarceration for a select geographic area. - Estimation of population-level prevalence of formerly incarcerated individuals by geographic area	Hysterectomy rates among incarcerated people. Gynecologic cancer familial risk screening and prevention in setting of disrupted families

### Civil rights laws and legal racial discrimination

#### Context in population health research

Legal racial discrimination has shaped access to resources, including mobility, income, employment, wealth, family and reproductive self-determination, and education since the founding documents of the United States.^25^ The 1964 U.S. Civil Rights Act (CRA) and other civil rights laws have changed the context in which racism operates, including by increasing access to resources (e.g., initiating reproductive and infant health services for Black people).^21,26^ Yet legal racial discrimination persists, as do the lingering effects of overturned laws. For example, any U.S.-born Black person 56 years of age and older in 2020 lived with Jim Crow laws, which mandated segregation of health care facilities, restricted access to health care, and provided federal funding of eugenics programs, which included coercive reproductive health procedures (e.g., forced sterilization). Immigration policy is another form of legal racial discrimination that carries out systemic racism by prohibiting access to citizenship and associated rewards to groups considered non-White. Such practices have appeared in the United States since the Naturalization Act of 1790.^4^ For any individual Black person living in the United States of any age, legalized racial discrimination and the remnants of overturned laws persist.^9,21^

#### Measures for use in population health studies

There is no single established measure of exposure to legal discrimination or its abolition. Time periods spanning the passage of civil rights laws and their enforcement mechanisms, or spanning the adoption or repeal of relevant immigration policies, along with meaningful geographic units, can be used to construct cohorts that serve as proxies. Research using age-period-cohort models shows improvement in life expectancy and premature mortality among Black populations born in the decade following passage of the CRA compared with the decade before^27^ and that Black women born in the late 1960s had a 70% decrease in adult health risk factors and improved birth outcomes compared with Black women born earlier that decade.^28^ Turning to geographic variation, estrogen receptor-negative breast cancer is significantly higher among Black women born in counties with active Jim Crow Laws, compared with peers born in counties without Jim Crow laws.^29^

The impact of variation in laws' implementation or enforcement can also be assessed using temporal or geographic cohorts. Hospitals' compliance with the 1965 Medicaid Act—which enforced Title VI of the CRA by tying funding to measures of integration—may have driven decreases in infant mortality.^28^ Infant health improvements during that time may have also been driven by implementation of maternal and child health funding under 1963 and 1965 mandates for health care provision to poor and Black populations.^30,31^

#### Applications to reproductive health

Measures of legal racial discrimination can be incorporated into a wide range of reproductive health research. Compulsory sterilization programs, for example, are salient to current reproductive health outcomes, including quality of contraceptive care delivery, mismatch between community preferences and current contraceptive offerings,^32^ and earned mistrust of the medical system, which may inhibit care-seeking behaviors, and embodiment of historical societal-level trauma. County-level associations between hysterectomy rate and race, independent of health care access, illustrate continued relevance of Jim Crow laws for gynecologic health.^33^ Black women—especially those in the U.S. South—remain at higher risk of premenopausal hysterectomy.^34^ Higher rates of hysterectomy is concerning in its own right, and may also limit community knowledge necessary to self-assess symptoms of gynecologic cancer, such as bleeding after menopause.^35^ The imprint of legalized discrimination as chronic stress may increase the burden of fibroids and endometriosis.^36^ Intergenerational effects may occur through cultural and institutional ideologies and practices, epigenetic^37^ and biological embodiment of trauma and stress,^38^ and durable maladaptive community beliefs and attitudes.^32,39^ Given these conceptual links, measures of legal racial discrimination—using age-period-birth cohorts and geography relative to the passage and enforcement of civil rights laws—can be incorporated into population reproductive health studies.

### Residential segregation and housing discrimination

#### Context in population health research

Racial residential segregation is the geographic separation of groups based on race. Segregation is a well-established determinant of adverse outcomes among Black populations in the United States.^40–43^ Mechanisms of segregation's impact on health include isolation, resource deprivation, and concentrating exposures to poverty, community violence, and environmental toxins.^40,43^ In addition, social network disruption can reduce the economic and social stability required to access health care and other health-promoting resources.^4,8^ Social networks may be disrupted due to constrained choice in housing due to displacement related to rising housing costs, housing discrimination, and the eradication of place-based subsidized housing toward “urban renewal” projects.^44^

Living in geographic areas characterized by racial and economic polarization is associated with higher rates of less treatable breast cancer subtypes,^45^ increased odds of hypertension,^46^ diabetes, obesity,^47^ premature mortality,^48^ and racial disparities cancer.^41^ Experiences of residential segregation early in life can have adverse consequences across the life course.^49^ Some studies illustrate a potential protective effect of living in racially homogenous areas, perhaps due to increased social cohesion where there is racial concordance between neighbors.^40,50^

Housing discrimination—including mortgage loan discrimination and redlining—has been used as measures of structural precedents of segregation.^50^ Race-based housing discrimination was outlawed with the Fair Housing Act in 1968,^50–52^ but housing discrimination and its health effects persist.^43^ Living in a neighborhood characterized by high racial bias in mortgage lending has been associated with increased all-cause mortality among Black women diagnosed with breast cancer^50,53^ and colorectal cancer.^53^ Residing in neighborhoods with a history of redlining increases vulnerability to declines in self-rated health.^51^

Experiences of residential segregation early in life have adverse health consequences across the life course, even after relocation,^49^ and these stressors persist across generations.^37^

#### Measures for use in population-based health studies

Racial bias in mortgage lending and redlining is captured with indices using spatially continuous estimates.^50^ The mortgage lending bias index represents the adjusted odds of home mortgage application denial for a Black compared to a White applicant within a geographic area. The redlining index represents the odds of denial of a mortgage application in particular areas compared with other areas.^50^ Both measures use data from the Home Mortgage Disclosure Act database.

Two summary metrics of the conditions created by segregation are prominent in the literature: The Area Deprivation Index (ADI) and the Index of Concentration at the Extremes (ICE). The ADI, which comprises 17 household-level socioeconomic indicators, describes the socioeconomic disadvantage of an area with high reliability and validity across census tracts.^54^ Although not explicitly a measure of racism-related variables, the ADI comprises indicators of conditions that can be conceptualized as downstream effects of systemic racism. Datasets at the state- and census-block levels using American Community Survey data for two timeframes in the 2000s are publicly available.^55^ The ICE quantifies the spatial feature of inequitable group relationships within an area and can be constructed using census data to describe racial or economic spatial inequity, or both in combination.^56^ The combined race and economic ICE outperforms measures using only race, income, or simple poverty rates in estimating the risk of health outcomes such as maternal health,^56^ and toxic environmental exposure.^57^ ICE is adaptable to different scales,^56^ which is useful for disentangling effects across geographic units.^4^

#### Applications to reproductive health

Multiple opportunities exist to investigate the role of residential segregation and housing discrimination in reproductive health outcomes. For example, while it is well documented that Black women residing in areas with higher disadvantage in the context of racialized and economic-based spatial inequity experience greater odds of infant mortality and preterm birth^18,58,59^; this pathway has been included in only a single study of gynecologic cancers and in no studies of benign gynecologic conditions of which we are aware. Promisingly, an association was found between higher scores on the ADI and cervical cancer incidence, late-stage diagnosis, and mortality rates, with a greater association for Black than White women.^60,61^ Using ADI and ICE in studies of gynecologic cancers^62^ and conditions such as fibroids^63^ would be a novel contribution to the literature. Including both measures would help to differentiate the roles of resource deprivation and inequity. Further upstream, mortgage loan bias and redlining could be used to examine the role of systematic disinvestment and segregation in these same outcomes within the life course and across generations.

Finally, qualitative research could supplement these quantitative measures to better understand the rich and complex role of social network formation and disruption in stress, gynecologic care delay, other mechanisms of harm, and coping responses.

### Police violence

#### Context in population health research

Police violence is a mechanism of structural racism that is of pressing public health concern.^8,64,65^ Today's institution of policing originated with slave patrols in the South, efforts to control immigrant populations in the North, and brutalization of Indigenous people across the continent. Its entrenchment has been bolstered by laws and policies that encourage overpolicing of Black neighborhood, reflect xenophobic and White supremacist ideologies, and criminalize behaviors more effectively addressed through public health intervention.^64^ Policing is enacted by policies and actions of police departments, federal law enforcement, and immigration enforcement (including immigration policies and enforcement practices). Physical and psychological violence occur through use of force, psychological intimidation, and community-level surveillance.^65–68^ Pathways to adverse health outcomes include direct injury and psychological harm, acute and chronic trauma and stress, and behavior change to limit exposure to policing, which inhibit access to health-promoting resources.^65–68^ Exposure to police violence also causes economic hardship due to costs of injury and judicial processes, or elimination of a household or community adult.^65^ Black people are at higher risk of death and nonfatal injury compared with White people.^65^ The adverse effects of police violence on Black people extend beyond direct encounters. In states with higher rates of police-involved deaths of unarmed Black people, Black people experience more days of mental health distress.^69^ Prevalence of asthma, diabetes, and high blood pressure is higher in neighborhoods where pedestrian stops are more likely to escalate into frisking, and where the frequency of such stops is higher among people of color.^70^

#### Measures in population-based studies of reproductive health

Efforts to incorporate measures of police violence into population health studies are made more difficult by limitations of official administrative databases: the Centers for Disease Control and Prevention's National Violent Death Reporting System, the U.S. National Vital Statistics System, and the Federal Bureau of Investigation's Uniform Crime Reporting System.^71,72^ Databases that combine reports from official sources, crowd-sourcing, news, and public records request account for the largest possible proportion of actual cases.^72^ For example, the Fatal Encounters database has been used to estimate county-level racial inequity in risk of homicide by police among males.^73^ State-level mental health effects have been investigated using the Mapping Police Violence database, linked to Behavioral Risk Factor Surveillance System data.^69^ Studies of health effects of immigration enforcement often use population surveys or administrative data from a timeframe before and after an immigration raid or policy change.^74,75^

The Survey of Police-Public Encounters captures self-reported police violence using a 27-item scale and has been conducted among a representative sample in four urban areas in the United States.^76^ Finally, the Jackson, Hogue, Philips contextualized stress measure assesses contextual and intersectional racial and gendered stress and includes two indicators of exposure to police violence.^77^ Using this measure, a positive association was found between antenatal depressive symptoms and anticipated negative youth encounters with police.^78^

#### Applications to reproductive health

Given the strength of association between stress and adverse reproductive health outcomes, and the documented association between psychological distress and police violence, it may be that police violence has far-reaching effects in reproductive health through early life, chronic, and intergenerational stress. This pathway is evident in studies finding adverse birth outcomes among Latinx parents following immigration raids or apprehensions,^74,79^ and for Latina, Middle Eastern/North African, and Arab women following changes in the social and political environment toward anti-immigrant sentiments.^80,81^ Anxiety related to constrained resources following an immigration raid has contributed to decisions to delay childbearing—demonstrating another stress pathway.^82^

To broaden this evidence to other forms of policing and across reproductive health domains, the Jackson, Hogue, Philips contextualized stress measure^78^ could be incorporated into population surveys investigating the role of stress not only in birth outcome but also in fibroids or histologic subtypes of gynecologic cancer, for example. Data from sources on policing exposure described above could be linked to nationally representative surveys that include mental health outcomes.

Limited mobility due to overpolicing of Black neighborhoods could also impact the ability to seek care for gynecologic health issues—abnormal uterine bleeding, pelvic pain from fibroids or endometriosis, and infertility. These conditions are associated with markedly worse burden and/or worse outcomes among Black women, but are not directly life threatening, so are particularly susceptible to care delays.

### Mass incarceration

#### Context in population health research

Mass incarceration in jails, prisons, and immigration detention centers is recognized as a steadily worsening mechanism of structural racism.^83,84^ Punitive policies and overpolicing of Black neighborhoods have resulted in the disproportionate impact of incarceration on Black people.^83^ Black children are five times more likely than White children to experience maternal incarceration.^85^ In 2006, nearly half of Black women had an immediate or extended family member imprisoned.^86^ Adverse health outcomes occur during incarceration and after release through policies and discrimination that inhibit access to housing, employment, and health care.^5,8,84^

Adverse health effects also occur among populations that are proximate to incarceration by relationship or geography. Parental incarceration is associated with asthma, behavioral issues, anxiety, depression, and worse overall self-rated health.^84^ Women whose partners are incarcerated are at greater risk for stroke, heart attack, and poor self-rated overall health.^87^ Higher rates of incarceration are associated with higher community-level prevalence of asthma and psychiatric morbidity.^84^ Community effects may occur because of financial strain; legal financial obligations place an immense burden on families and communities, and are often shouldered by women.^88^ Other mechanisms include reduced social support due to the removal of community members, incarceration-related stigma, or forced displacement; political disenfranchisement; and higher levels of both acute and chronic stress.

#### Measures for use in population health studies

Linking administrative incarceration data with population-level health metrics provides the most accurate method by which to estimate health effects and inequities.^84^ State--level rates of incarceration can be estimated using Public Use Microdata Samples data, which are collected at the decennial census and contain flags for sample members who are institutionalized.^84,89,90^ A more nuanced estimation of all current and formerly incarcerated individuals to assess community-level effects can be constructed^90^ using a combination of data sources from the U.S. Department of Justice and U.S. Bureau of Prisons.^84,90^

#### Applications to reproductive health

Studies that have leveraged state-level data to study the effects of incarceration on reproductive health have documented higher incidence of infant mortality, poorer access to or quality of prenatal care,^91^ risk of contracting infections disease,^92^ and racial disparities within these outcomes.^84^ Parental incarceration is associated with increased risk of infant mortality and increases in inflammatory markers among female children^85^—well documented as part of the pathway connecting pre-conception stress with adverse pregnancy outcomes.^93^ Coercive contraceptive practices and family separation are also more likely to occur in the context of mass incarceration.^68,91^

These measures might be meaningfully extended to studies of reproductive health outcomes beyond maternal and infant outcomes. Pathways for future investigation include the role of mass incarceration in (1) care delay, due to limited social support or economic strain, which may contribute to later-stage diagnosis of gynecologic cancers; or (2) physiologic stress mechanisms that promote more aggressive gynecologic disease.^36,62,63^ In addition, access to care for any of these conditions may be hindered by fear of incarceration due to punitive approaches to behaviors such as drug use, or to conditions such as homelessness or immigration status, all of which are better addressed through an approach that prioritizes addressing structural causes of these conditions.

### Multidomain measures

Employing multidimensional measures of structural racism may be useful to understand the interlocking and compounding harms of its various mechanisms, which may otherwise be obfuscated when using only discrete measures. Lukachko et al.^94^ identify four domains for measuring state-level variation in exposure to structural racism: the Black–White relative proportions of political participation, employment and job status, educational attainment, and judicial treatment.^94^ Data for the index are drawn from the U.S. Census Bureau; National Conference of State Legislatures; Bureau of Labor Statistics; and the Sentencing Project.^94^ Using this index, higher levels of structural racism have been associated with higher odds of small-for-gestational-age birth^95^ and higher rates of infant mortality^96^ among Black residents. This measure could be feasibly applied across the spectrum of reproductive health outcomes.

### Measures for future development

There are other mechanisms of structural racism that impact health, for which there are not yet quantitative measures.^21^ For example, Black people are disproportionately exposed to the child welfare system compared with other racial and ethnic groups.^97,98^ Exposure to foster care can adversely affect adult health outcomes in a cumulative manner, contributing to higher rates of hypertension, asthma, diabetes, cardiovascular risk, chronic health conditions, stroke, and epilepsy,^99,100^ as well as STDs.^99,101^ Although no national data are available, there are several large-scale studies that capture foster care involvement and could be linked with reproductive health data. The California Health Interview Survey is the largest statewide health survey administered in the United States. Several regional studies also exist, including the Midwest Evaluation of the Adult Functioning of Former Foster Youth.^101^

The literature we reviewed largely excludes trans and nonbinary individuals whose reproductive and gynecologic health may be uniquely affected by the exposures described above. Developing measures of structural racism that uniquely effect populations with marginalized gender and sexual identities will help better assess the underlying, interlocking mechanisms of harm, and candidate intervention foci.^22^

Indeed, intersectionality—a concept coined by Kimberlé Crenshaw—is core to the implementation of a reproductive justice framework, and therefore, intersecting identities are a necessary focus of population reproductive health research.^102^ Having a cognitive or physical disability, for example, which is disproportionately more likely among Black people,^103^ increases risk of exposure to violent policing and exacerbates health consequences of direct or community-level exposure.^104^ The stress pathways described for each factor above are likely made worse in the case of existing disability. Immigration status is another dimension that warrants attention. The policies, practices, and ideologies of a racialized system of immigration enforcement that targets Black people and people of color act as forms of legalized racial discrimination, as mechanisms of policing and incarceration, and drive residential segregation.^4, 66–68^ Others note the need to understand the intersection of race, nativity, and immigration status in health research,^4,102,105,106^ and emerging research describes the salience of this intersection for maternal and infant health outcomes.^107–109^

## Discussion

Measures of exposure to systemic racism are important to include in population-based studies of reproductive health. The existing evidence suggests there are many plausible hypotheses to be investigated beyond those related to maternal and infant health. We reviewed some of the many domains of systemic racism that engender and exacerbate adverse reproductive health outcomes among Black women in the United States, described existing measures of these domains, and presented opportunities for applying them in population reproductive health studies. Importantly, we focused on systemic racism. When we limit our understanding of the effects of racism to interpersonal and psychosocial pathways, we reinforce the notion that race and racism occur at the individual level and we obfuscate and absolve the sociopolitical structure of racism. The carefully constructed scaffolding of interlocking systems and ideologies that reinforce the racialized distribution of resources is intentionally hidden from view, not least by limiting the means to measure it. Systemic racism requires our explicit, empirical attention to identify effective means to deconstruct it and its contributions to racial inequities in reproductive health. Efforts to advance equity in reproductive health require population health research that explicitly incorporates measures of systemic racism.

As a fundamental cause of health inequities, systemic racism constantly replaces the mechanisms that ensure unequal distribution of power, prestige, social connections, money, and freedom.^5,23^ Examining and intervening on a single domain or considering domains as static will not reduce racial inequities in reproductive health.^23^ It is recommended that researchers not only include multiple measures in historical context but also constantly reassess how measures must be altered to capture the iterative transformation of mechanisms of harm.

Our review corroborates previous recommendations to take a life course perspective that examines mechanisms including social network formation, early childhood stress, and compounding stress,^110^ and to integrate epigenetic mechanisms.^111,112^ Finally, while our focus is systemic racism in the United States, our domain and framework could be adapted for other contexts.

Efforts to improve reproductive health outcomes among Black women require population health research that explicitly incorporates measures of systemic racism. Our review illustrates conceptual and methodological paths toward that end.

## Abbreviations Used

ADI: Area Deprivation Index
CRA: Civil Rights Act
ICE: Index of Concentration at the Extremes
